# Fluorescent ratiometric supramolecular tandem assays for phosphatase and phytase enzymes†

**DOI:** 10.1039/d3ob02014b

**Published:** 2024-01-29

**Authors:** Kirk M. Atkinson, Bradley D. Smith

**Affiliations:** a Department of Chemistry and Biochemistry, 251 Nieuwland Science Hall, University of Notre Dame IN 46556 USA smith.115@nd.edu

## Abstract

Ratiometric fluorescent assays have a built-in correction factor which enhances assay accuracy and reliability. We have developed fluorescent ratiometric supramolecular tandem assays for phosphatase and phytase enzymes using a mixture of three molecular components. One of the molecules is a tetra-cationic fluorescence quencher called CalixPyr which can bind and quench the polyanionic pyrene fluorophore, CMP, that emits at 430 nm. Polyphosphates can disrupt the CMP/CalixPyr complex and alter the fluorescence intensity (responsive signal). CalixPyr has no effect on the fluorescence emission of cationic pentamethine cyanine fluorophore, cCy5, which emits at 665 nm and acts as a non-responsive reference signal. The continuous ratiometric fluorescent assay for alkaline phosphatase monitored hydrolytic consumption of adenosine triphosphate (ATP). The continuous ratiometric fluorescent assay for phytase activity monitored hydrolytic consumption of phytate. With further development this latter assay may be useful for high throughput assessment of phytase activity in individual batches of fortified animal feed. It is likely that the three-molecule mixture (CMP, CalixPyr, cCy5) can become a general assay platform for other enzymes that catalyse addition/removal of phosphate groups from appropriate molecular substrates.

## Abstract 2

Two continuous ratiometric fluorescent assays for phosphate hydrolase enzymes are created based on selective binding and quenching of a responsive visible dye in the presence of a non-responsive deep-red reference dye.

## Graphical abstract

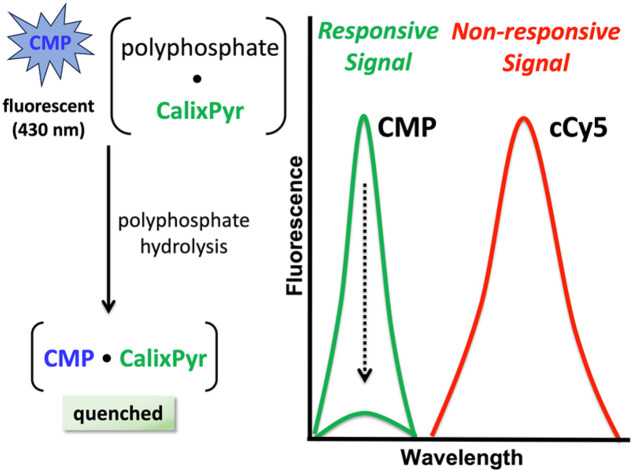

## Introduction

One of the major technical achievements of supramolecular chemistry over the last twenty years is the development of indicator displacement assays.^1^ Fluorescent versions of these assays employ a paired combination of fluorophore and host molecule that have the capacity to reversibly associate and form a non-covalent complex with modified fluorescence signal. In the presence of a competing analyte the mole fraction of fluorophore/host complex is altered and so is the corresponding fluorescence signal. A popular manifestation of the fluorophore displacement strategy is the supramolecular tandem assay (STA) which is used to monitor enzymatic reactions that modulate the concentration of a competing analyte over time.^2–4^ Typically, these assays track the change in fluorescence intensity at a single wavelength (intensiometric detection); however, it is well-established that a more reliable assay design tracks a responsive signal relative to a non-responsive reference signal at a separate wavelength (ratiometric detection).^5,6^ Thus, there is a need for generalizable methods of converting an intensiometric fluorescent STA into a ratiometric STA. The simplest idea is to add a second reference fluorophore to the responsive fluorophore/host mixture. The reference fluorophore must possess a specific set of properties including: (a) an emission wavelength that can be detected separately from the wavelength of the responsive fluorophore, and (b) negligible affinity for the other molecules in the assay mixture. Here, we describe a new supramolecular strategy for converting an intensiometric STA into a ratiometric STA.

**Scheme 1:**
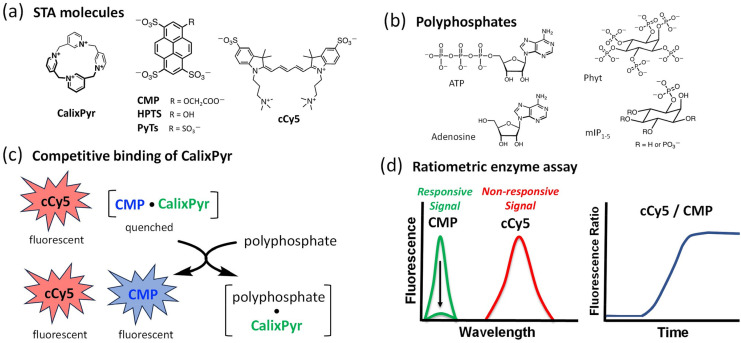
(a) Chemical structures of the STA molecules used in this study. (b) Polyphosphates used in this study. (c) Selective binding and quenching of CMP fluorescence by CalixPyr is modulated by the presence of polyphosphate. (d) Ratiometric assay reports time-dependent change in fluorescence signals for non-responsive reference cCy5 and responsive CMP which can be plotted as the cCy5/CalixPyr fluorescence ratio.

The host molecule in this study is calixpyridinium (CalixPyr), a tetra-cationic molecule that was invented by Tsukube and co-workers in 1998 (Scheme 1a).^7^ Over the intervening years, CalixPyr has been incorporated into various supramolecular assembly paradigms.^8–19^ A notable property of CalixPyr is its capacity to complex and quench the fluorescence of polysulfonated pyrene fluorophores such as HTPS and PyTs. Moreover, the quenching efficiency can be lowered by the presence of competing polyphosphates or polycarboxylates that have affinity for the tetra-cationic CalixPyr.^20–22^ This has led to the exploitation of fluorophore/CalixPyr pairs within intensiometric enzyme assays that track ATP hydrolysis by phosphatase enzymes.^23^ While the commercial availability of HTPS and PyTs is an attraction, the pH sensitivity of HTPS,^24^ and the low excitation wavelength of PyTs (340 nm which is below the common diode laser wavelength range) are drawbacks that limit the scope of enzyme assays that can be developed. We reasoned that a ratiometric and pH-insensitive fluorescent STA could be achieved by combining CalixPyr in a mixture with two new fluorophores that have a unique set of spectral and supramolecular properties. The first new fluorophore is the polyanionic pyrene derivative CMP which exhibits a pH-insensitive emission at 430 nm.^25^ We hypothesized that CalixPyr would bind to polyanionic CMP and quench its fluorescence, and if so CMP would be a superior replacement for HTPS or PyTs as a CalixPyr-responsive fluorophore within an STA. The second new fluorophore is the non-responsive reference fluorophore cCy5 with 665 nm emission. The chemical structure of cCy5 has a spatially wide distribution of opposite charges and a net positive charge, thus we hypothesized that it would exhibit negligible affinity for CalixPyr. We have confirmed both hypotheses by conducting fluorescence and NMR titration studies that show CalixPyr can selectively complex and quench CMP in the presence of cCy5. Moreover, we find that competitive polyphosphate binding to CalixPyr (Scheme 1c) selectively modulates the CMP fluorescence signal and enables us to exploit this three-molecule mixture (CMP, CalixPyr, cCy5) as a fluorescent ratiometric STA (Scheme 1d) for two separate enzymes that remove phosphate groups from polyphosphate molecules, specifically the enzymatic conversion of ATP to adenosine, and the enzymatic conversion of phytate (Phyt) to *myo*-inositol-phosphate_1–5_ (mIP_1–5_) (Scheme 1b).

## Results and discussion

### Molecular design and compound synthesis

CMP, CalixPyr, and cCy5 were synthesized by adapting literature procedures as outlined in the ESI.† It is worth emphasizing that CalixPyr is prepared in one step from commercially available 2-bromomethyl pyridine and that it exhibits very low cell toxicity.^26^ The fluorophore CMP is a member of the Cascade Blue family of pyrene fluorophores which are known to be very bright and photostable.^27^CMP is tetra-anionic at neutral pH, it can be excited by a common 405 nm diode laser and its fluorescence spectrum with 430 nm maxima is invariant above pH 4.^25^cCy5 is a deep-red pentamethine cyanine dye with absorbance and emission maxima peaks at 644 nm and 665 nm respectively.

CalixPyr affinity and selectivity for CMP was measured by conducting a series of fluorescence titration experiments that added aliquots of CalixPyr to separate solutions of CMP and monitoring the decrease in CMP fluorescence intensity at 430 nm. Each titration isotherm fitted nicely to a 1 : 1 binding model (Fig. S1–S5†) and the derived association constants (*K*_a_) are listed in Table 1. The *K*_a_ of 1.3 × 10^5^ M^−1^ for the formation of CalixPyr·CMP in water, pH 6.8 is comparable to the reported *K*_a_ values for CalixPyr binding to HTPS or PyTs in water.^20–22^ The measured *K*_a_ values were somewhat higher in biological buffers, (Table 1) but there was a substantial decrease, due to electrostatic screening, when a high concentration of potassium phosphate was used as the buffer.^28^

**Table 1:** Association constants for formation of CalixPyr·CMP in different buffers

Buffer	Association constant (*K*_a_)	pH
Water	(1.3 ± 0.1) × 10^5^ M^−1^	6.8
10 mM NaOAc	(1.3 ± 0.2) × 10^6^ M^−1^	7.2
5 mM TES	(3.6 ± 0.8) × 10^6^ M^−1^	7.2
10 mM HEPES	(3.0 ± 1.0) × 10^6^ M^−1^	7.0
100 mM K-Phos	(3.1 ± 1.8) × 10^3^ M^−1^	7.0

**Fig. 1:**
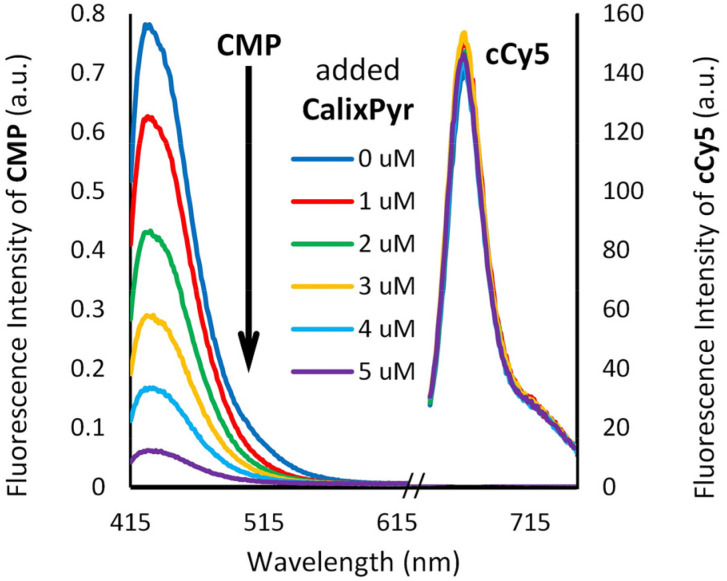
Fluorescence spectra for a single solution containing a binary mixture of CMP (5 μM) and cCy5 (5 μM) in water (pH 6.8) and titrated with CalixPyr. *λ*_ex_ = 401 nm (CMP) and 630 nm (cCy5), slit width = 1 nm.

The capacity of CalixPyr to selectively quench CMP in the presence of cCy5 was demonstrated by titration experiments that added aliquots of CalixPyr to a solution containing a binary mixture of CMP and cCy5. As shown by the fluorescence spectra in Fig. 1 the titration produced selective quenching of CMP fluorescence intensity at 430 nm and negligible change in cCy5 fluorescence at 665 nm.

### ^1^H NMR titration

**Fig. 2:**
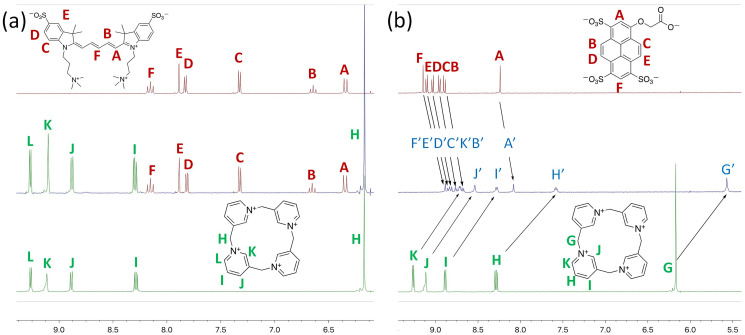
(a) Partial ^1^H NMR spectrum (500 MHz, D_2_O, pD 6.63, 25 °C) of cCy5 [1 mM] (top), CalixPyr [1 mM] + cCy5 [1 mM] (middle), and CalixPyr [1 mM] (bottom). (b) Partial ^1^H NMR spectrum (500 MHz, D_2_O, pD 6.63, 25 °C) of CMP [1 mM] (top), CalixPyr [0.5 mM] + CMP [1 mM] (middle), and CalixPyr [1 mM] (bottom).

^1^H NMR spectroscopy provided confirmation of CalixPyr binding selectivity for CMP over cCy5. Shown in Fig. 2 are comparisons of NMR spectra for binary mixtures of CalixPyr with CMP or with cCy5. The spectra in Fig. 2a show that mixing CalixPyr with cCy5 at a 1 : 1 ratio produces no change in chemical shift for any of the peaks, strongly indicating no measurable interaction. In contrast, the spectra in Fig. 2b clearly show that mixing CalixPyr with CMP at a 0.5 : 1 ratio produces large up field changes in chemical shift for both species, indicating formation of a CalixPyr·CMP complex that is in rapid exchange with the free molecular species (see expanded NMR spectra in Fig. S7 and S8†). When the CalixPyr·CMP ratio was increased to 1 : 1 (1 mM each compound) there was substantial broadening of the peaks and formation of yellow aggregates (Fig. S6†) seen by the naked eye. Complex precipitation is not surprising since a 1 : 1 complex of tetra-cationic CalixPyr and tetra-cationic CMP has a net neutral charge and a moderately hydrophobic aromatic surface.

### CMP/CalixPyr fluorescence selectivity for ATP

**Fig. 3:**
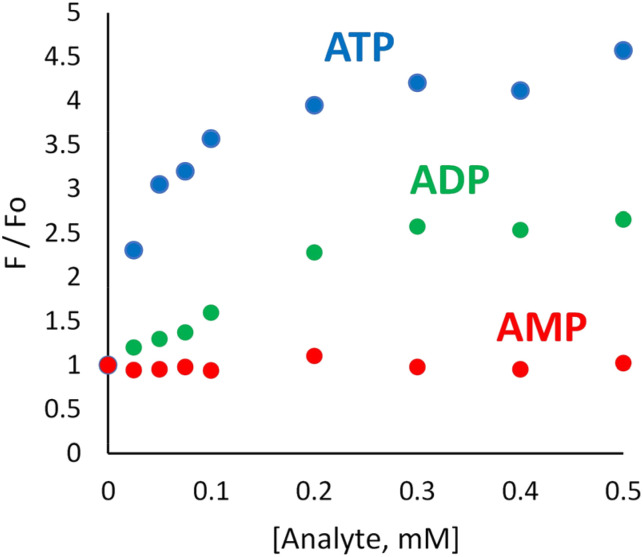
Change in fluorescence intensity (*F*/*F*_0_) for CMP (*λ*_ex_ = 401 nm, *λ*_em_ = 430 nm) upon incremental addition of ATP, ADP, or AMP to separate solutions containing CMP (1 μM), cCy5 (1 μM) and CalixPyr (4 μM) in 10 mM NaOAc solution, pH 7.2, room temperature.

The association constant between CMP and CalixPyr was determined to be 1.3 × 10^6^ M^−1^ at pH 7.2 in 10 mM NaOAc solution (Table 1). Previous studies using the same buffer have found that tetra-cationic CalixPyr has affinity for adenine nucleotides in the order ATP (5.0 × 10^4^ M^−1^) > ADP (1.4 × 10^4^ M^−1^) > AMP (4.8 × 10^2^ M^−1^) which reflects the differences in nucleotide anionic charge. This trend suggested to us that the complementary pair of CMP and CalixPyr can be used to create an ATP-selective indicator displacement assay.^23^ Experimental confirmation was gained by conducting titration experiments that added ATP, ADP, or AMP to separate solutions containing CMP (1 μM), cCy5 (1 μM), and CalixPyr (4 μM) in 10 mM NaOAc solution at pH 7.2. The titration isotherms in Fig. 3 show that the order of fluorescence response (*i.e.*, increase in CMP fluorescence intensity at 430 nm) was ATP > ADP > AMP. Moreover, the titrations induced no change in the cCy5 fluorescence intensity (Fig. S12†) indicating that the ratio of the two fluorescence signals (*i.e.*, cCy5/CMP) could be monitored as a ratiometric response. In the following sections, we describe how this three-molecule mixture (CMP, CalixPyr, cCy5) was developed as a fluorescent ratiometric STA for two different phosphate hydrolase enzymes (Scheme 1d).

### Alkaline phosphatase assay

**Fig. 4:**
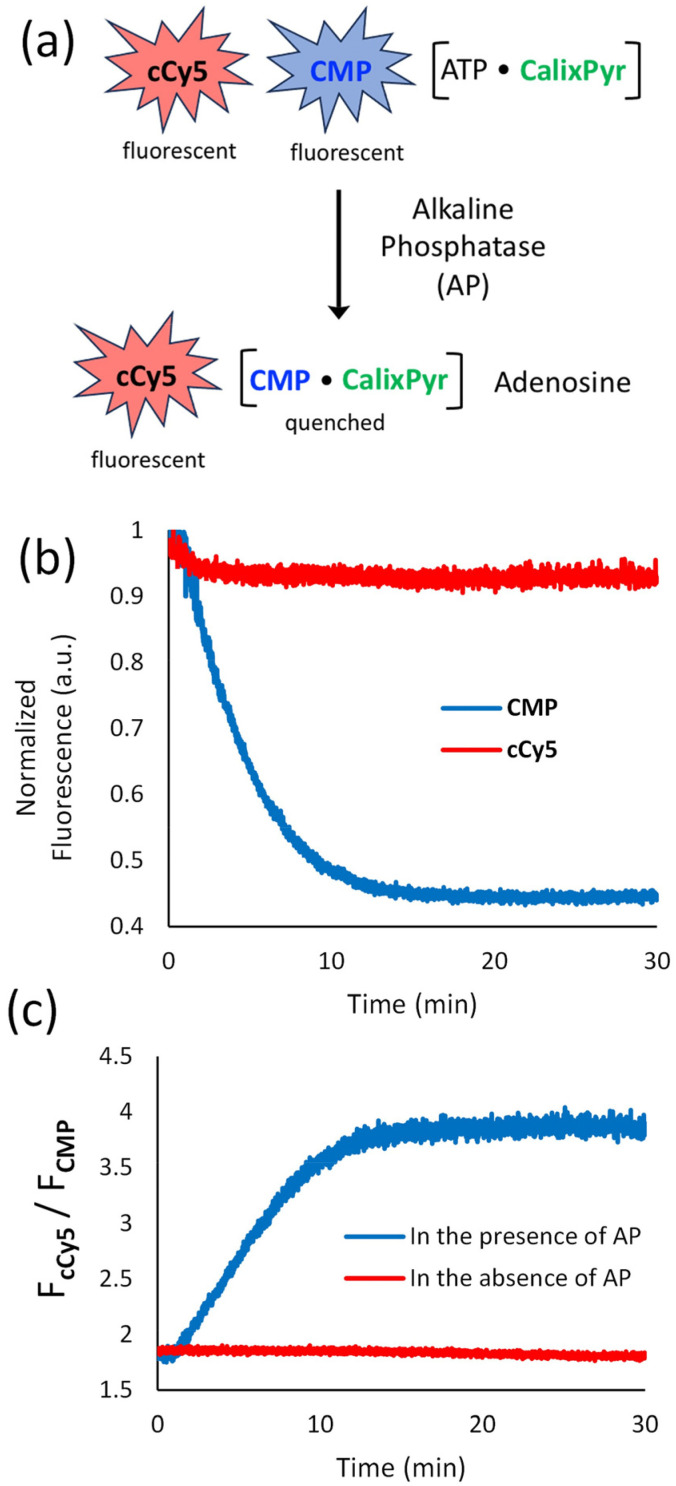
(a) Summary of continuous ratiometric fluorescent assay that monitors consumption of ATP catalysed by AP. (b) Typical data set showing change in normalized CMP and cCy5 fluorescence after addition of AP (1.5 U mL^−1^) at 1 min, also in the assay mixture was CalixPyr (4 μM), CMP (1 μM), cCy5 (1 μM), ATP (60 μM), 10 mM NaOAc, pH 7.2, 37 °C. (c) Same assay data set plotted as the ratio of fluorescence intensities, *F*_cCy5_/*F*_CMP_, in the presence and absence of AP, (CMP, *λ*_ex_ = 401 nm, *λ*_em_ 430 nm; cCy5, *λ*_ex_ = 630 nm, *λ*_em_ 665 nm).

We first used the three-molecule mixture (CMP, CalixPyr, cCy5) to create an STA for alkaline phosphatase (AP), a ubiquitous enzyme that can hydrolyse ATP.^23^ High levels of alkaline phosphatase in clinical fluids such as serum are indicators of health problems such as Pegat's disease, breast and prostate cancer, vitamin D deficiency, or liver damage.^29^ Robust high throughput assays of AP activity are also needed in drug discovery programs that aim to identify and characterize AP inhibitors.^30^ The literature contains many fluorescence assays that track consumption of ATP but only a small fraction of them produce a ratiometric response.^4,31–37^ In Fig. 4a is a schematic summary of our continuous ratiometric AP assay. At time zero, CalixPyr is bound by polyanionic ATP and thus cannot bind and quench the fluorescence of CMP. Addition of AP catalyses sequential removal of phosphate groups from the ATP to eventually produce adenosine. Consumption of ATP enables the CalixPyr to selectively bind CMP and quench its fluorescence signal at 430 nm and not the cCy5 signal at 665 nm (Fig. 4b and S13†). The data is replotted in Fig. 4c as a time-dependent change in the ratio of fluorescence intensities, *F*_cCy5_/*F*_CMP_.

### Phytase assay

The AP assay described above was conducted in a buffer of 10 mM NaOAc solution at pH 7.2. Most likely at this pH, the assay could have successfully employed the literature fluorescent dye HTPS instead of CMP.^20^ But the pH sensitivity of HTPS (p*K*_a_ ***∼ 7.3) prevents its use in the second STA we developed, which measures the activity of the enzyme phytase in weak acid.

Phytate (Phyt, Scheme 1b) is the conjugate base of phytic acid, and it is stored within plant seeds as salts with the phosphate groups strongly chelating biologically important minerals such as calcium, iron, or zinc. While Phyt has high nutritional value, it is not readily digested by humans or monogastric livestock such as pigs and poultry.^38^ Not only is there the problem of untapped nutritional potential, but the excreted Phyt leads to elevated phosphorus concentrations in manure and contributes to surface water eutrophication.^39^ Phytases are a group of phosphate hydrolase enzymes that remove phosphate groups from Phyt, and they are widely employed as feed supplements to enhance Phyt nutrition and to reduce phosphate discharge into the environment.^39–41^ Phytase catalyses sequential hydrolytic removal of phosphate groups from Phyt leading to the formation of lower *myo*-inositol-phosphates_1–5_ (mIP_1–5_, Scheme 1b) which reduces metal-binding affinity and enhances mineral bioavailability. The nutritional and environmental benefits of phytase in animal feed and agricultural produce provides substantial motivation to develop rapid and accurate analytical methods to quantify phytase content in individual batches of whole grain and legume feedstocks.^42–44^ The most commonly employed analytical methods measure the amount of released inorganic phosphate but sometimes this does not produce a sufficiently accurate assessment of actual phytase activity. Recently, discontinuous methods that measure loss of Phyt have been developed based on ion chromatography or lateral flow strips, but they are resource intensive and likely to be costly on a large scale.^45,46^ There are surprisingly few fluorescent sensing systems that report Phyt levels,^47–50^ and there appears to be no published example of a ratiometric fluorescent assay for phytase activity.^51–53^

**Fig. 5:**
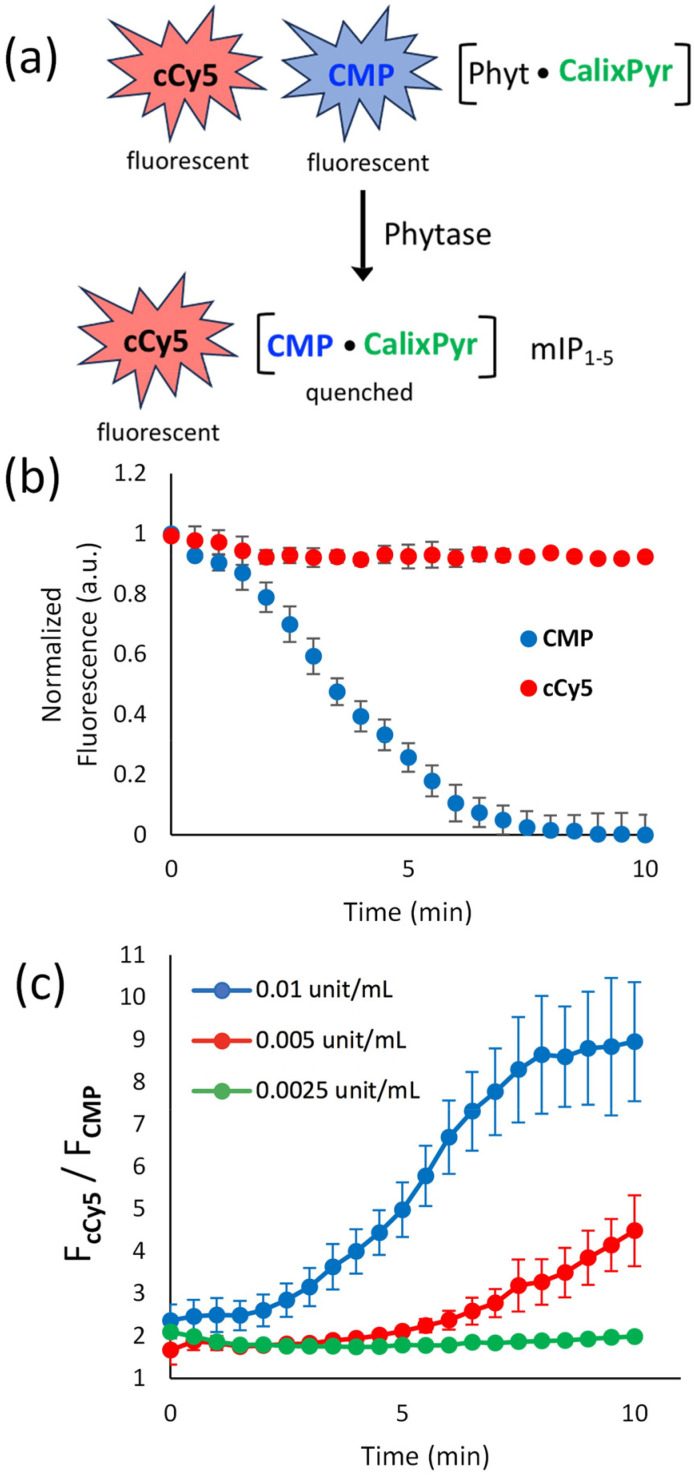
(a) Summary of continuous ratiometric fluorescent assay that monitors consumption of Phyt catalysed by phytase. (b) Typical data set showing change in normalized CMP and cCy5 fluorescence after addition of phytase (0.01 U mL^−1^) at time zero the assay mixture contained CalixPyr (4 μM), CMP (1 μM), cCy5 (1 μM), and phytate (100 μM) in 5 mM HEPES buffer, pH 5.1 at 45 °C. (c) Ratio of fluorescence intensities, *F*_cCy5_/*F*_CMP_, over time for samples containing different of phytase levels, (CMP, *λ*_ex_ = 401 nm, *λ*_em_ 430 nm; cCy5, *λ*_ex_ = 630 nm, *λ*_em_ 665 nm).

To demonstrate proof of concept we developed the continuous ratiometric phytase assay that is summarized in Fig. 5a. A series of titration experiments proved that Phyt could bind to CalixPyr under acidic conditions (pH 5.1, 5 mM HEPES buffer) and prevent quenching of CMP (Fig. S9†). A competitive titration experiment (Fig. S10 and S11†) determined the association constant between Phyt and CalixPyr to be 2.0 × 10^6^ M^−1^ at pH 5.1 and an additional fluorescence experiment revealed that Phyt has no effect on cCy5 fluorescence intensity. This suggested that the STA in Fig. 5a was feasible. At assay time zero, CalixPyr is bound by polyanionic Phyt and cannot bind and quench the fluorescence of CMP. Addition of phytase induces sequential removal of phosphate groups from the Phyt and production of mIP_1–5_. The consumption of Phyt allows the CalixPyr to bind and selectively quench the CMP signal at 430 nm and not the cCy5 signal at 665 nm (Fig. 5b). The profiles in Fig. 5c show change in the ratio of fluorescence intensities, *F*_cCy5_/*F*_CMP_ over time. An assay sample containing 0.005 units per mL of phytase produced an observable change in ratiometric signal after ten minutes but there was no measurable response when the phytase activity was 0.0025 units per mL, thus reflecting the assay's limit of detection. An unusual feature of the assay response profile is the initial lag phase, and this kinetic feature is attributed to the multistep chemical and supramolecular process. When the assay begins, all the inositol molecules in the sample are Phyt which has a −6 charge and binds to the tetra-cationic CalixPyr. As the Phyt phosphate groups are removed by phytase catalysed hydrolysis there is sequential conversion of highly anionic mIP_1–5_ species to less anionic mIP_1–5_ species that have reduced affinity for CalixPyr.‡‡The affinity of CalixPyr for the different mIP_1–5_ species is not known, and it is not clear how quickly affinity decreases as the mIP_1–5_ species become less anionic. Most likely, the assay lag time could be reduced by optimizing the concentration of assay components. After about two minutes (when phytase is 0.01 unit per mL) CalixPyr binding of tetra-anionic CMP becomes favourable and there is an increasing amount of CMP fluorescence quenching.

## Conclusions

Fluorescent ratiometric supramolecular tandem assays for phosphatase and phytase enzymes were developed using the three-molecule mixture (CMP, CalixPyr, cCy5). The common supramolecular features of each assay are: (a) tetra-cationic CalixPyr can bind and quench the polyanionic pyrene derivative CMP which exhibits a pH-insensitive emission at 430 nm. (b) Polyphosphates can disrupt the CMP/CalixPyr interaction and alter the fluorescence intensity (responsive signal). (c) CalixPyr has no effect on the fluorescence emission of cationic cCy5 at 665 nm and so it can be used as a non-responsive reference signal. Demonstration of utility was first gained by developing a continuous ratiometric fluorescent assay for alkaline phosphatase (AP) catalysed hydrolysis of ATP. A more innovative and impactful result is development of a continuous ratiometric fluorescent assay that monitors consumption of Phyt catalysed by phytase. It is likely that this three-molecule mixture can be deployed as a ratiometric supramolecular tandem assay for other enzymes that catalyse addition/removal of phosphate groups from appropriate molecular substrates.^4,31–37^

## Experimental

Polyphosphates, reagents and solvents were purchased from Sigma-Aldrich, BioChemika, VWR, Oakwood, Thermo Fisher, Ambeed or TCI and used without further purification unless stated otherwise. Calf intestinal alkaline phosphatase was purchased from Invitrogen, catalogue number: 18009019. Phytase from wheat was purchased from Sigma-Aldrich, catalogue number: P1259. The reagent solutions were freshly prepared daily. The pH and pD values were verified on a Mettler Toledo Benchtop F20 pH mV^−1^ Standard Kit pH meter calibrated with three standard buffer solutions. The pH readings were converted to pD by adding 0.4 units.

### Synthesis and characterization

All synthetic procedures and compound characterization are provided in the ESI.†

### Titration methods

Direct titrations measured the association of CMP and CalixPyr in different aqueous solutions. A solution of CMP (1 mL) was placed in a cuvette and aliquots of CalixPyr solution were added to the CMP solution, keeping the [CMP] constant. Spectral changes were recorded after each aliquot addition. A plot of fluorescence intensity for CMP at 430 nm was fitted to a 1 : 1 binding model, as described in the ESI.†

The association of Phyt and CalixPyr was measured by a competitive titration method. A solution of CMP and CalixPyr (1 : 4 ratio, 1 mL) was placed in a cuvette and aliquots of Phyt solution were added, keeping the [CMP/CalixPyr] constant. A plot of CMP fluorescence intensity at 430 nm was fitted to a 1 : 1 competitive binding model, as described in the ESI.†

### Enzyme cuvette studies

Separate stock solutions of CMP, CalixPyr, cCy5, Phyt, ATP, were prepared in ultrapure deionized water or the designated buffer. Aliquots of CMP, CalixPyr, cCy5, Phyt, ATP were added to cuvettes containing the designated buffer and were warmed to the appropriate temperature. AP or phytase was added and the solutions (2.0 mL) were mixed by stir bar while changes in the fluorescence spectra were measured using a spectrometer in time-scan mode. Iterative sequential time scan cycles scanned CMP and then cCy5 for a total acquisition time of 2 seconds, followed by 28 seconds interval before the next data point. Enzyme assays were repeated in triplicate. These assays employed a spectrometer, but they should be amenable to a plate reader.

## Conflicts of interest

The authors declare no competing financial interest.

## Supplementary Material

OB-022-D3OB02014B-s001
