# Supplementary material for: Fluorescent ratiometric supramolecular tandem assays for phosphatase and phytase enzymes
Source: Org Biomol Chem. 2024 Jan 29;22(8):1714–20. doi: 10.1039/d3ob02014b (PMC10911839; doi:10.1039/d3ob02014b)
Supplement: OB-022-D3OB02014B-s001 [file OB-022-D3OB02014B-s001.pdf]

Electronic Supplementary Material (ESI) for Organic & Biomolecular Chemistry.
This journal is © The Royal Society of Chemistry 2024

Electronic Supplementary Information for:

Fluorescent ratiometric supramolecular tandem assays for phosphatase and phytase enzymes

Kirk M. Atkinson and Bradley D. Smith*

General

Column chromatography was performed using Biotage Sfär columns. ^{1}H and NMR spectra were recorded on a Bruker 400 or 500 NMR spectrometer. Chemical shifts are presented in ppm and referenced by residual solvent peak. High-resolution mass spectrometry (HRMS) was performed using a time-of-flight (TOF) analyzer with electrospray ionization (ESI). Absorption spectra were recorded on an Evolution 201 UV/vis spectrometer with Thermo Insight software. Fluorescence spectra were collected on a Horiba Fluoromax Plus fluorometer and FluoroEssence software. All absorption and fluorescence spectra were collected using quartz cuvettes (1 mL, 1 cm path length).

Synthesis

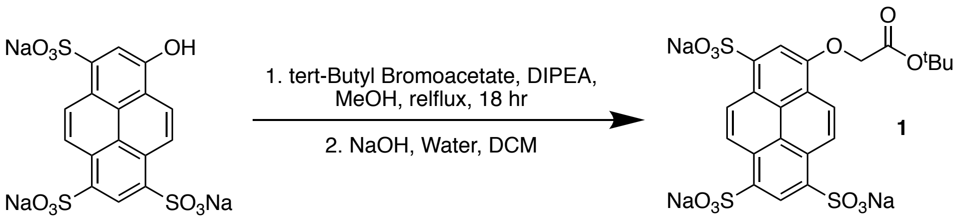

Compound 1. A solution of pyranine (515 mg, 0.97 mmol, 1 eq), DIPEA (420 µL, 2.41 mmol, 2.48 eq), tert-butyl bromoacetate (635 mg, 3.24 mmol, 3.33 eq) and 50 mL of MeOH was refluxed 16 hr. The reaction mixture was vacuum filtered, and the filtrate was evaporated. The resulting crude was purified by reverse phase column chromatography (C18, 0-50% MeOH in H_2O) to produce a pale-yellow solid. The crude material was dissolved in water with an equimolar equivalent of NaOH and was extracted 3 times with DCM. The aqueous layer was evaporated and further purified by reverse phase column chromatography (C18, 0-50% MeOH in H_2O) to produce pure compound **1** as a pale-yellow solid (271.3 mg, 43.7%).^{1}H NMR (400 MHz, D_2O) δ 9.17 (s, 1H), 9.06 (d, *J* = 9.8 Hz, 1H), 8.96 (d, *J* = 9.6 Hz, 1H), 8.91 (d, *J* = 9.8 Hz, 1H), 8.63 (d, *J* = 9.6 Hz, 1H), 8.07 (s, 1H), 4.96 (s, 2H), 1.32 (s, 9H).

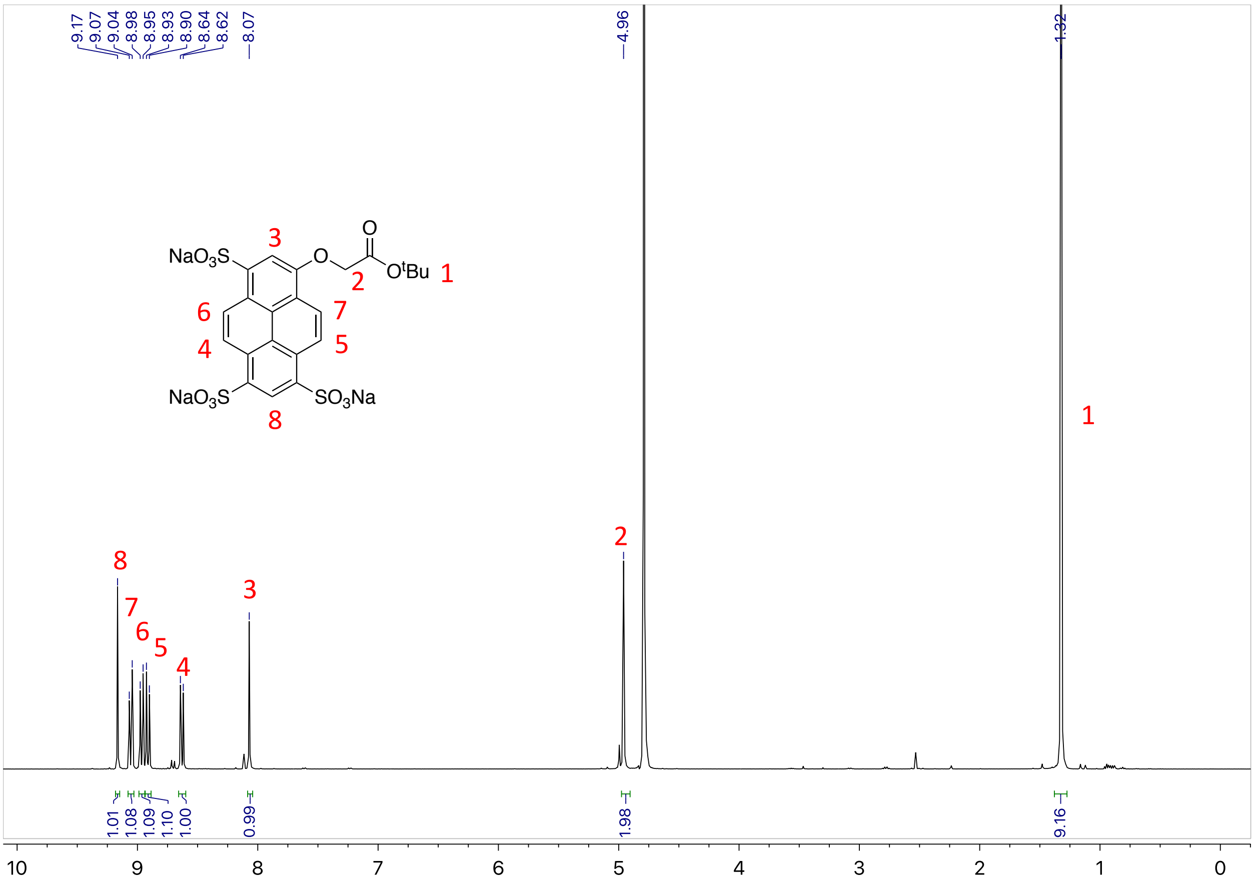

^{1}H NMR spectrum (400 MHz, D_2O) of **compound 1**.

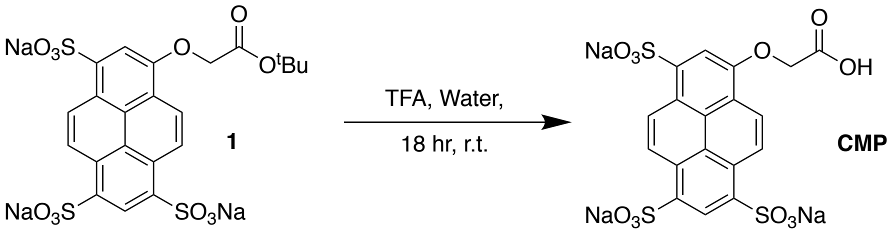

CMP. A solution of 20 mL of water, compound **1** (271.3 mg, 0.425 mmol, 1 eq), and 2 mL of TFA was stirred at room temperature for 18 hr. The reaction mixture was then evaporated to yield the known compound **CMP** [1] as a pale-yellow powder (231.7 mg, 93.6%). ^{1}H NMR (400 MHz, D_2O) δ 9.06 (s, 1H), 8.98 (d, *J* = 9.8 Hz, 1H), 8.89 (d, *J* = 9.6 Hz, 1H), 8.83 (d, *J* = 9.8 Hz, 1H), 8.64 (d, *J* = 9.6 Hz, 1H), 8.07 (s, 1H), 5.03 (s, 2H).

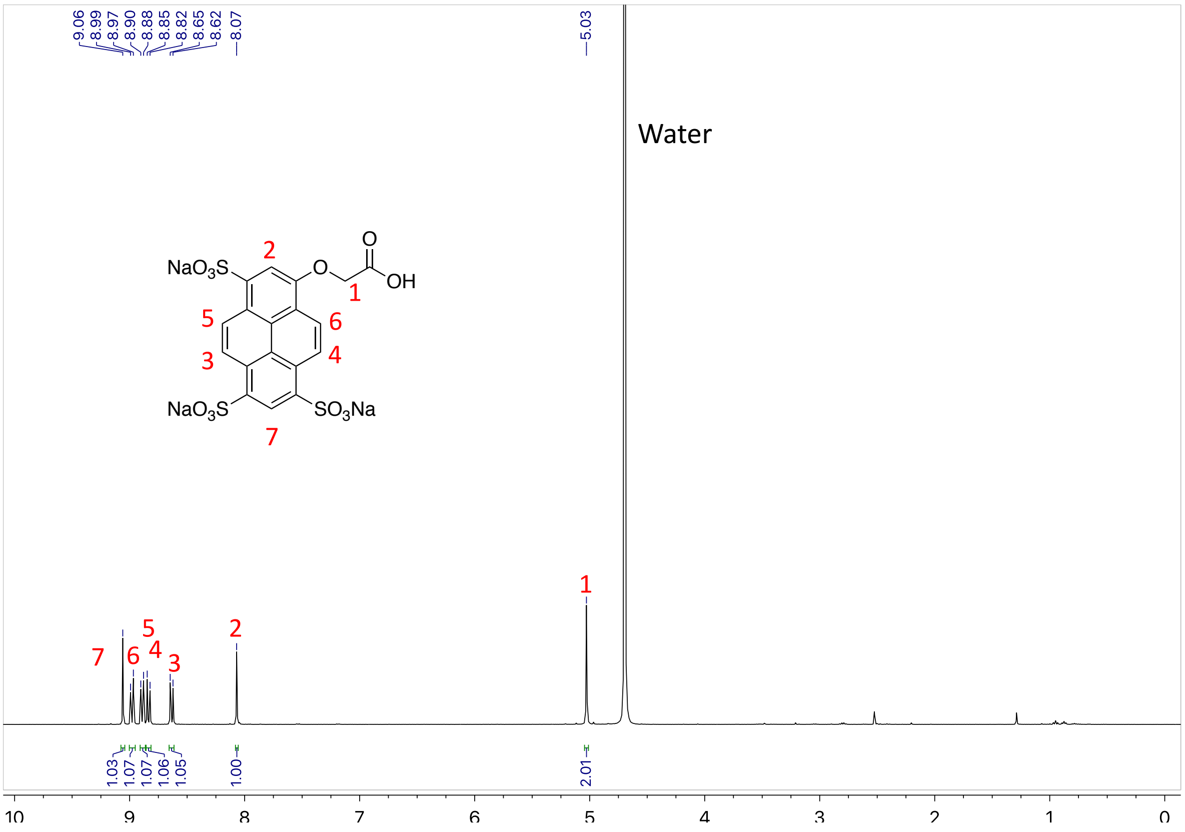

^{1}H NMR spectrum (400 MHz, D_2O) of **CMP**

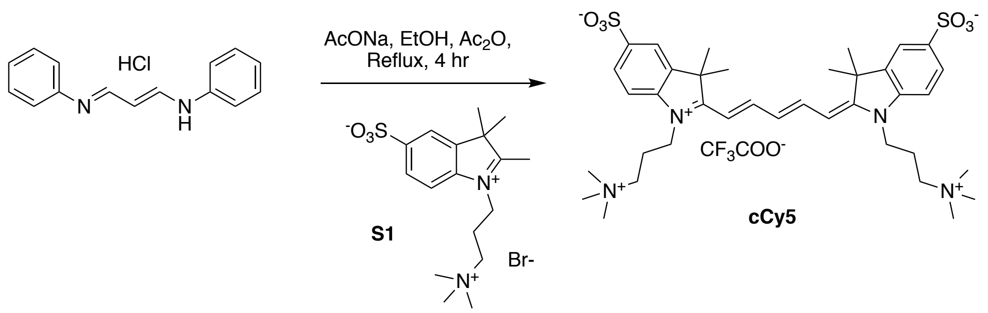

cCy5. A solution of N-(3-(phenylamino)-2-propenylidene) aniline hydrochloride (82 mg, 0.3 mmol, 1 eq), indolenine **S1**[2] (~80 wt%, 475 mg, 0.9 mmol, 3 eq), AcONa (172 mg, 2.1 mmol, 7 eq), 5 mL of Ac_2O, and 5 mL of EtOH was refluxed at 100˚ for 4 hr. The reaction was stopped, cooled to room temperature and 35 mL of Et_2O was added and stored at -20˚ for 20 min. The blue precipitate was vacuum filtered and purified by reverse phase column chromatography (C18, 0-40% MeOH (0.5% TFA) in H_2O) to afford pure **cCy5** as a blue solid (202.2 mg, 80.4%). ^{1}H NMR (400 MHz, D_2O) δ 8.12 (t, *J* = 13.1 Hz, 2H), 7.86 (d, *J* = 1.6 Hz, 2H), 7.79 (dd, *J* = 8.4, 1.7 Hz, 2H), 7.28 (d, *J* = 8.4 Hz, 2H), 6.62 (t, *J* = 12.5 Hz, 1H), 6.32 (d, *J* = 13.6 Hz, 2H), 4.16 (t, *J* = 7.0 Hz, 4H), 3.50 – 3.43 (m, 4H), 3.10 (s, 18H), 2.30 (dt, *J* = 15.4, 7.6 Hz, 4H), 1.67 (s, 12H). ^{13}C NMR (101 MHz, D_2O) δ 174.39, 163.89, 159.79, 155.44, 143.82, 141.67, 139.17, 126.55, 119.89, 110.57, 104.25, 63.13, 52.92, 49.13, 26.79, 20.78. HRMS (ESI+): calcd for $C_{37}H_{53}N_4O_6S_2$ $[M + H]^+$ 713.3401, found 713.3391.

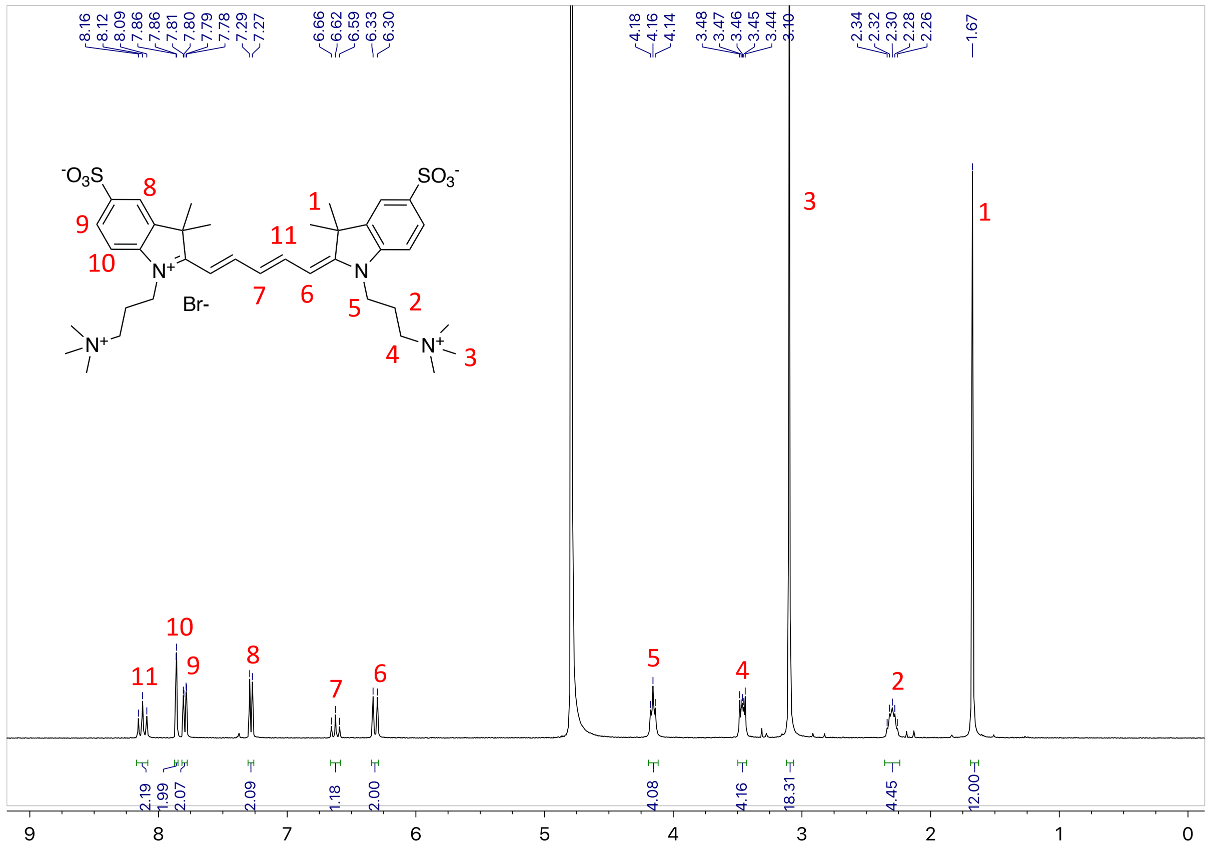

^{1}H NMR spectrum (400 MHz, D_2O) of **cCy5**

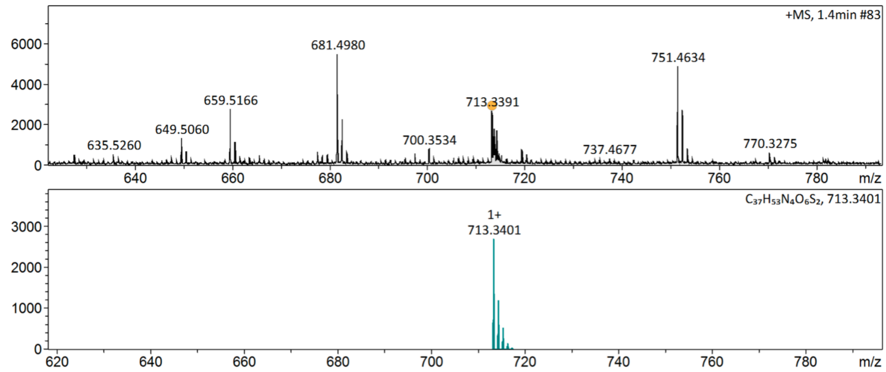

HRMS (ESI-TOF) spectrum of **cCy5**

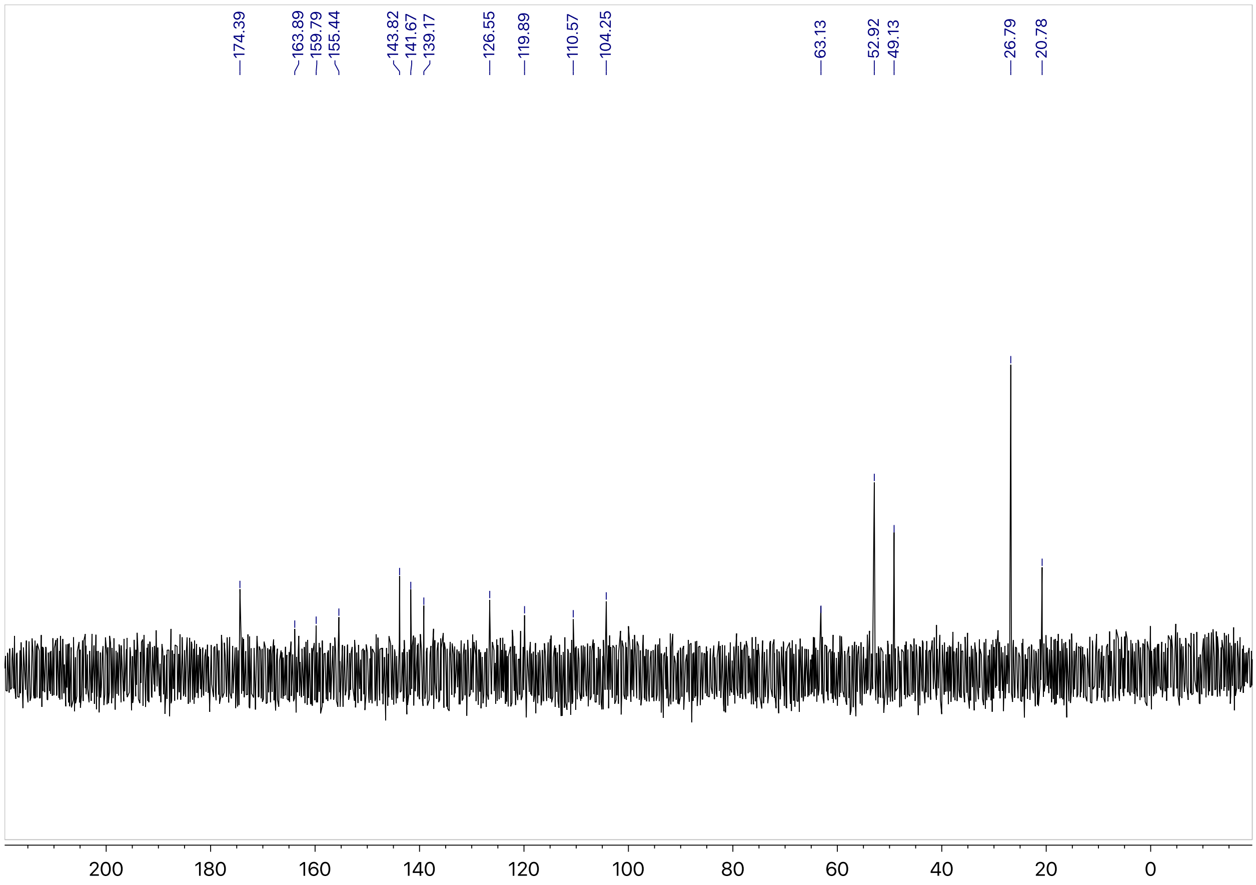

^{13}C NMR spectrum (101 MHz, D_2O) of **cCy5**

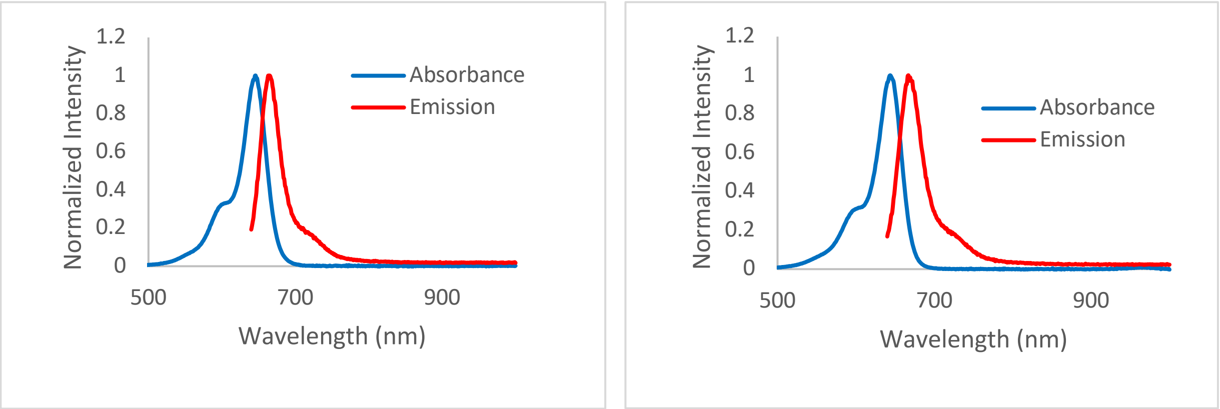

Absorbance and emission spectra of **cCy5** (5 µM) in methanol (left) and water (right), λ_{ex} = 630 nm, slit width = 1 nm.

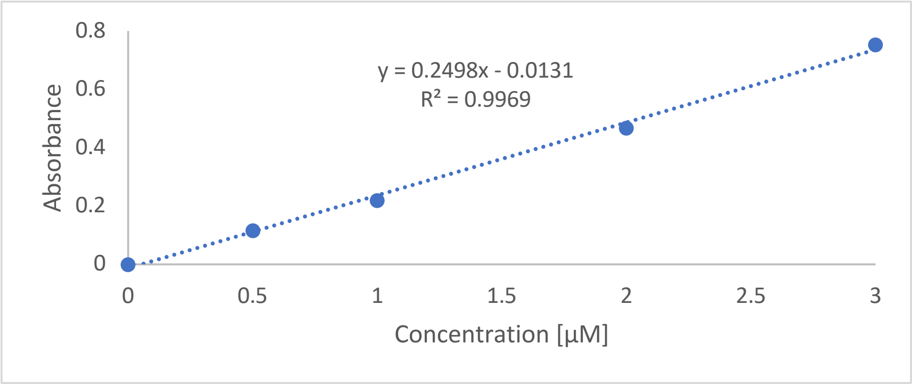

Dependence of absorption at λ_{max} on the concentration of **cCy5** in MeOH.

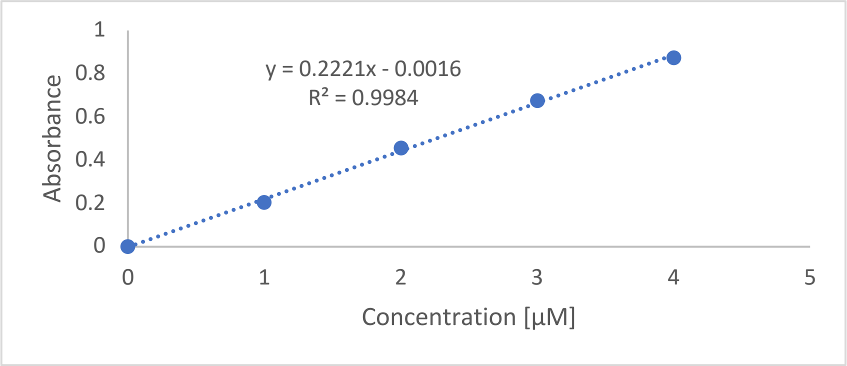

Dependence of absorption at λ_{max} on the concentration of **cCy5** in water.

	**CMP**	**cCy5**
ϕ_f[a]	0.96[b]	0.41
ε (L.mol^{-1}cm^{-1})	25,780	222,100
Brightness[3]	24,749	91,061

Table S1. Photophysical characterization of **CMP** and **cCy5** in water at room temperature. [a] Absolute fluorescence quantum yield was measured directly by photon counting, error is ± 5%. [b] ref 1. [c] Brightness = ε × ϕ_F, error is ± 15%.

Fluorescence quantum yield measurements of cCy5

Absolute quantum yield of **cCy5** was measured on a Horiba Fluoromax Plus spectrometer with an integrating sphere. Samples were excited at 630 nm with absorbance ≤ 0.05. The photons were recorded with an integrating sphere upon excitation of a blank solvent reference, then the reference was replaced by a sample solution, and the spectrum 620 – 800 nm was acquired. The quantum yield was calculated by the equation below:

$$\phi_F = \frac{P_{em}}{P_{abs}} = \frac{\int_B^A (F_{sample} - F_{blank})\, d\lambda}{\int_D^C (E_{blank} - E_{sample}) d\lambda}$$

where *P* is the number of photons, *F* is the fluorescence intensity and *E* is the intensity at the excitation wavelength. Experiments were conducted in triplicate at three different concentrations, with the reported absolute quantum yields corresponding to the mean value, and ± 5% systematic error was determined.

cCy5 (λ_{ex} = 630 nm) : A = 800, B = 640, C = 640 nm, D = 620 nm. $\phi_F = 33.8\%$ in methanol and 40.5% in water.

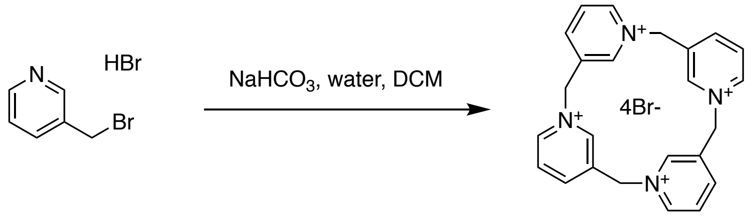

CalixPyr. Synthesized according to the literature.[3] ^{1}H NMR (400 MHz, D_2O) δ 9.45 (s, 1H), 9.25 (d, *J* = 6.1 Hz, 1H), 8.89 (d, *J* = 8.2 Hz, 1H), 8.27 (t, *J* = 8.2 Hz, 8.0 1H), 6.19 (s, 2H).

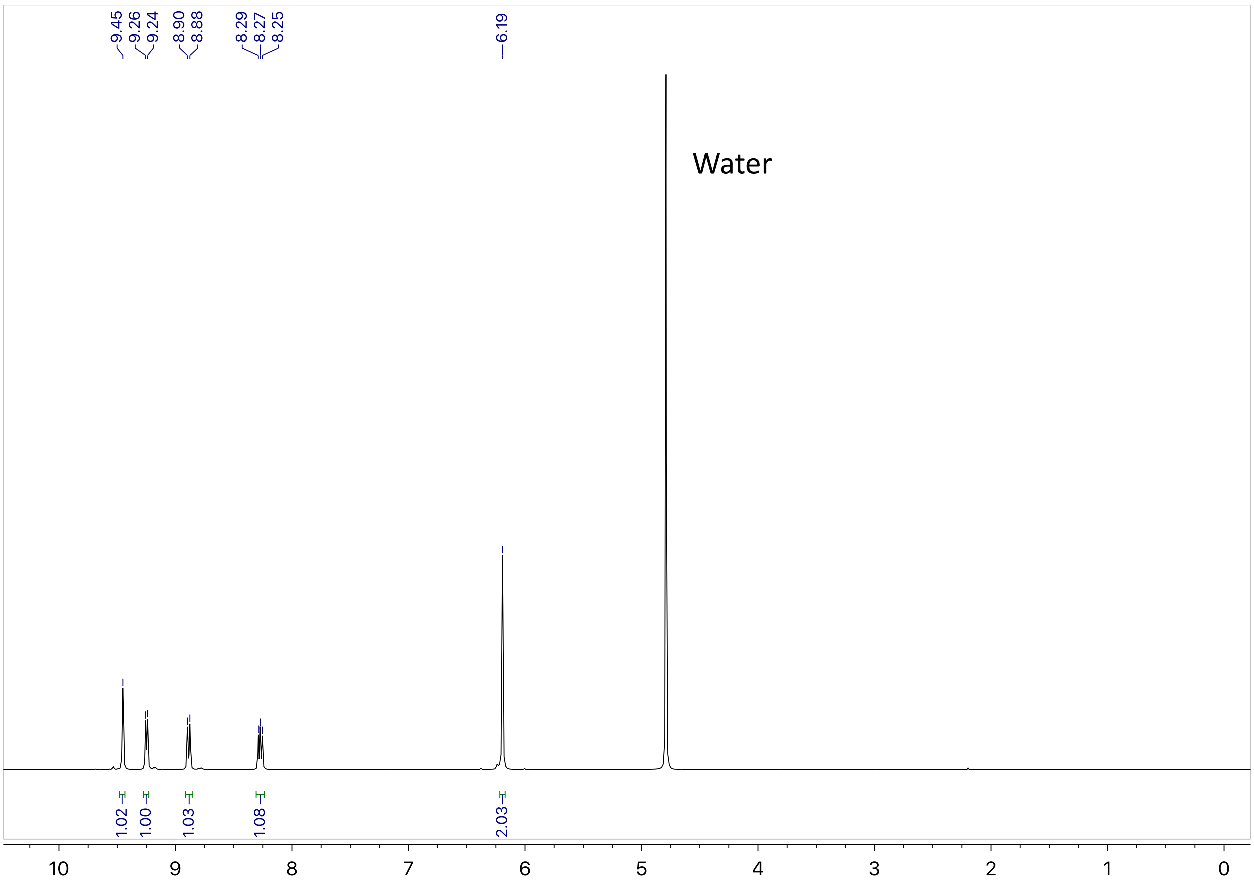

^{1}H NMR spectrum (400 MHz, D_2O) of **CalixPyr**

CMP and Calixpyridinium association constant in different aqueous buffers (direct titration)

For a 1:1 host-guest system:

$$H + G \underset{}{\overset{K_a}{\rightleftharpoons}} HG$$

The desired association constant is expressed in equation (1.1):

$$K_a = \frac{[HG]}{[H][G]} \quad (1.1)$$

Assigning the total concentration of host and guest as $[H]_0$ and $[G]_0$, respectively, gives mass balance equations (1.2) and (1.3):

$[H]_0 = [H] + [HG]$ (1.2)

$[G]_0 = [G] + [HG]$ (1.3)

Equation (1.2) is rearranged to define [H] and equation (1.3) is rearranged to define [G]. The newly defined [H] and [G] are used to replace [H] and [G] in equation (1.1) and the resulting equation is rearranged to yield equation (1.4):

$[HG]^2 - ([H]_0 + [G]_0 + 1/K_a) [HG] + [H]_0[G]_0 = 0$ (1.4)

The real root of equation (1.4) is expressed in equation (1.5), which defines [HG] based on K_a and experimentally determined values ($[H]_0$ and $[G]_0$):

$$[HG] = 0.5\left(\left\{[H]_0 + [G]_0 + \frac{1}{K_a}\right\} - \sqrt{\left([H]_0 + [G]_0 + \frac{1}{K_a}\right)^2 - 4[H]_0[G]_0}\right) \quad (1.5)$$

The association constant was calculated form the titration data with specifically written non-linear least squares fitting the equation for 1:1 binding within Origin Lab™ 8.6 software.

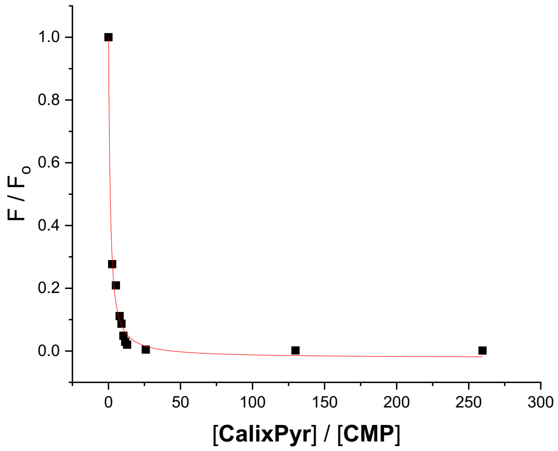

Figure S1 Association constant for **CalixPyr** and **CMP** in water (pH 6.8), λ_{ex} = 401 nm, slit width 1 nm. $K_a = (1.3 \pm 0.1) \times 10^5$ M^{-1}. $R^2 = 0.992$.

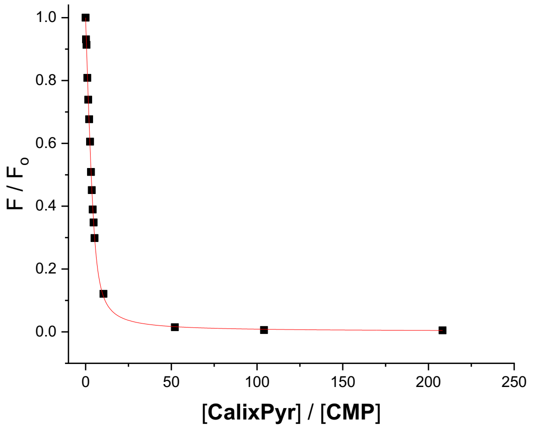

Figure S2 Association constant for **CalixPyr** and **CMP** in 10 mM NaOAc (pH 7.2), λ_{ex} = 401 nm, slit width 1 nm. $K_a = (1.3 \pm 0.2) \times 10^6$ M^{-1}. $R^2 = 0.999$.

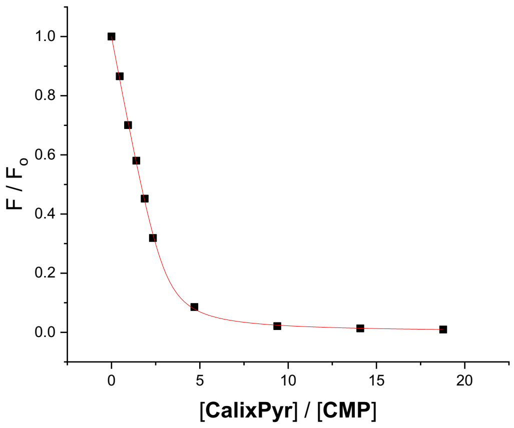

Figure S3 Association constant for **CalixPyr** and **CMP** in 5 mM TES buffer (pH 7.2), λ_{ex} = 401 nm, slit width 1 nm. K_a = (3.6 ± 0.8) × 10^6 M^{-1}. R^2 = 0.999.

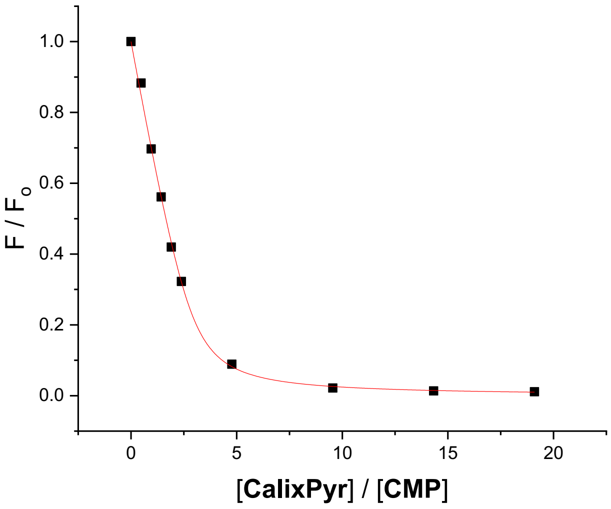

Figure S4 Association constant for **CalixPyr** and **CMP** in 10 mM HEPES buffer (pH 7.0), λ_{ex} = 401 nm, slit width 1 nm. K_a = (3.0 ± 1.0) × 10^6 M^{-}1. R^2 = 0.998.

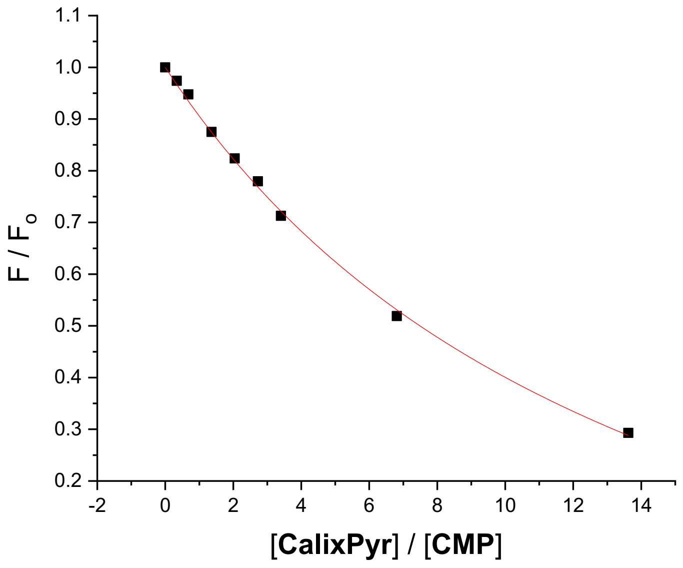

Figure S5 Association constant for **CalixPyr** and **CMP** in 100 mM potassium phosphate buffer (pH 7.0), λ_{ex} = 401 nm, slit width 1 nm. K_a = (3.1 ± 1.8)×10^3 M^{-1}. R^2 = 0.998.

NMR Experiments

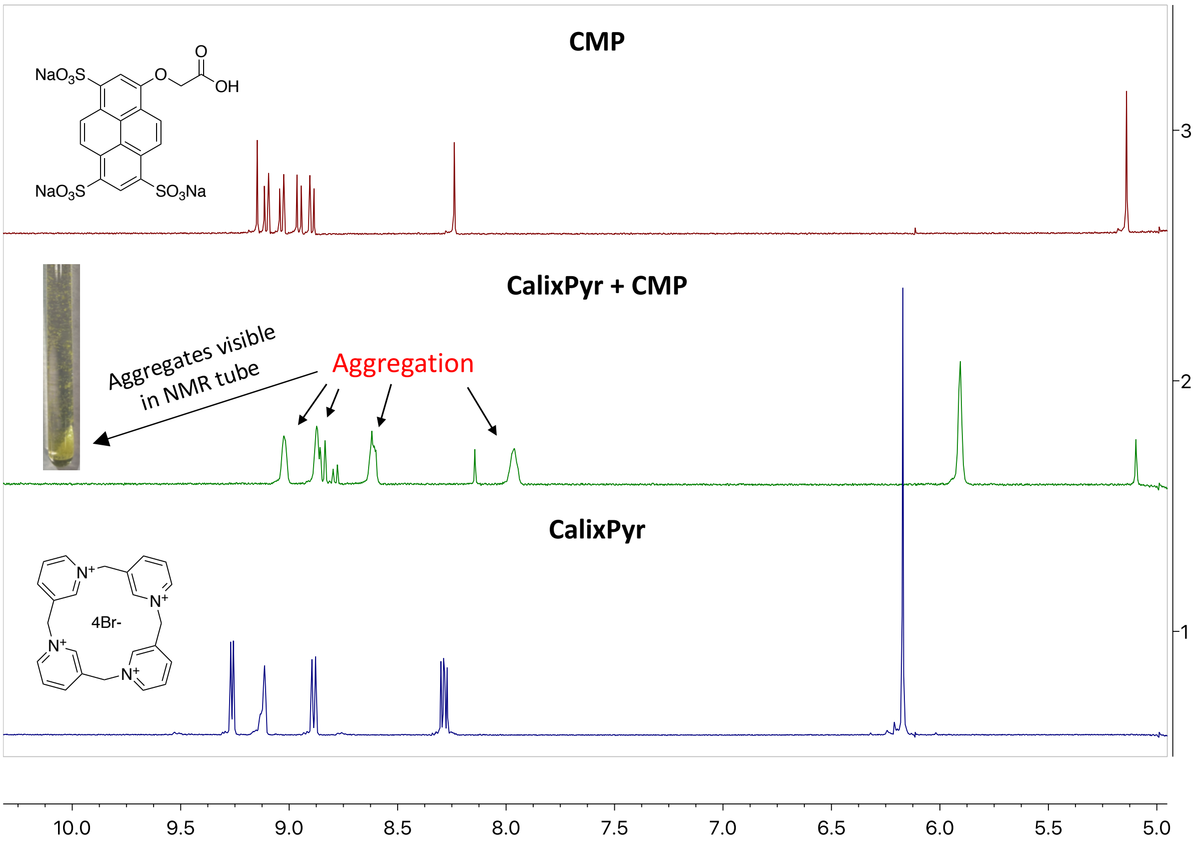

Figure S6 Partial ^{1}H NMR spectrum (500 MHz, D_2O, pD 6.63, 25 °C) of **CMP** (1 mM, sodium salt), **CalixPyr** (1 mM)•**CMP** (1 mM), and **CalixPyr** (1 mM).

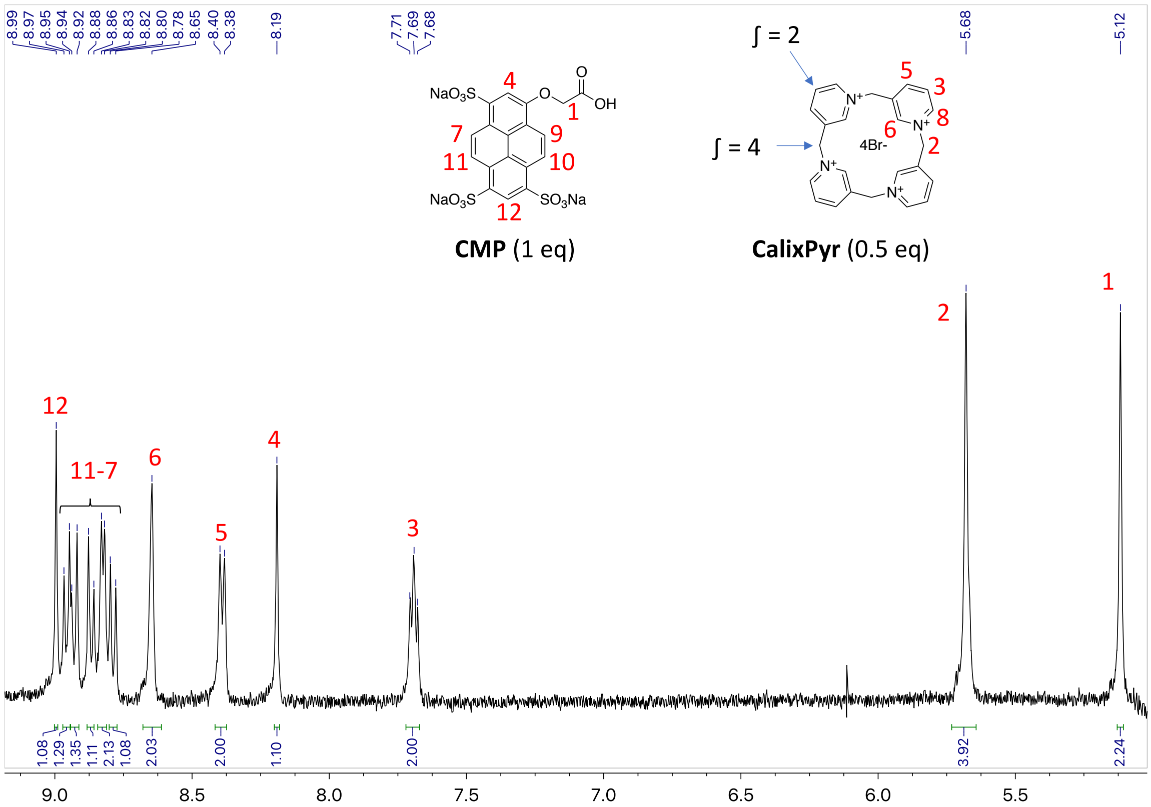

Figure S7 Partial ^{1}H NMR spectrum (500 MHz, D_2O, pD 6.63, 25 °C) of **CalixPyr** (0.5 mM)•**CMP** (1 mM).

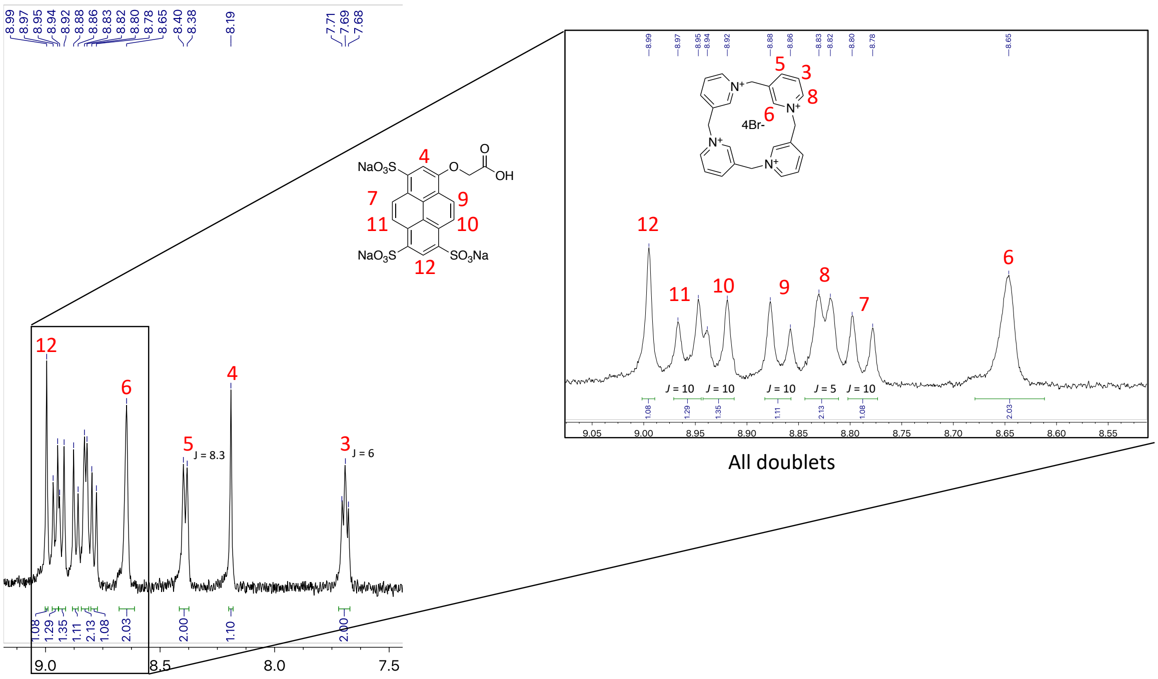

Figure S8 Expanded ^{1}H NMR spectrum (500 MHz, D_2O, pD 6.63, 25 °C) for **CalixPyr** (0.5 mM)•**CMP** (1 mM).

Phytate Titration

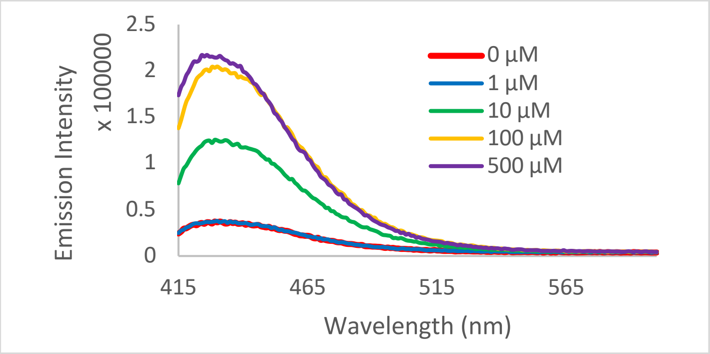

Figure S9 Emission spectra of **CMP** (1 µM) with increasing concentration of Phyt (λ_{ex} = 401 nm, slit width = 1 nm). Also present in the solution is **cCy5** (1 µM), **CalixPyr** (4 µM), in 5 mM HEPES (pH 5.1)

Competitive titration of Phyt in the presense of **CalixPyr** and **CMP**

In the following equation derivation, we will use H as **CalixPyr**, I as **CMP**, and G as Phyt. For the competitive binding, we have

$$HI + G \rightleftharpoons HG + I$$

The equilibrium between H and G is:

$$H + G \overset{K_a}{\rightleftharpoons} HG$$

$$K_a = \frac{[H][G]}{[HG]} \quad (2.1)$$

The equilibrium between H and I is:

$$H + I \overset{K_i}{\rightleftharpoons} HI$$

$$K_i = \frac{[H][I]}{[HI]} \quad (2.2)$$

According to mass balance equation,

$$[H]_t = [H] + [HG] + [HI] \quad (2.3)$$

$$[G]_t = [G] + [HG] \quad (2.4)$$

$$[I]_t = [I] + [HI] \quad (2.5)$$

Now we will derive an equation involving only one unknown concentration. Here we use [H] and focus on equation (2.3). We will seek to define all other concentrations to define [H], accordingly, equation (2.1)-(2.5) can be rearranged to

$$[HG]=\frac{K_a[H][G]_t}{1+K_a[H]}$$ (2.6)

$$[HI]=\frac{K_i[H][I]_t}{1+K_i[H]}$$ (2.7)

$$[I]=\frac{[I]_t}{1+K_i[H]}$$ (2.8)

Substitute (2.6)-(2.8) to (2.3) yielded

$$[H]_t=[H]+\frac{K_a[H][G]_t}{1+K_a[H]}+\frac{K_i[H][I]_t}{1+K_i[H]}$$ (2.9)

Equation (2.9) is a cubic equation for [H] and can be rearranged as

$$A[H]^3+B[H]^2+C[H]+D=0$$ (2.10)

$$A=K_iK_a$$

$$B=K_i+K_a+K_iK_a[I]_t+K_iK_a[G]_t-K_iK_a[H]_t$$

$$C=1+K_i[I]_t+K_a[G]_t-K_i[H]_t-K_a[H]_t$$

$$D=-[H]_t$$

In the competitive titration experiment, Phyt was titrated into **CalixPyr•CMP** complex (4:1 molar ratio). We treated the **CalixPyr•CMP** complex as non-fluorescent while the **CalixPyr•**Phyt complex (HG) as fluorescent. The relation between measured fluorescence signals with the real concentration of HG can be written as

$$F=k[HG]$$ (2.11)

k was the emission coefficient

We combine equation (2.6) and (2.11) to give the final equation

$$F=\frac{k\times K_a\times[H]\times[G]_t}{1+K_a\times[H]}$$ (2.12)

Equation (2.12) was used for nonlinear fitting using Origin Lab™ software version 8.6. In the above equation, $[G]_t$ was the total concentration of Phyt for each titration and Newton's method was used to iterate the real concentration of **CalixPyr** ([H]) from equation (2.10).

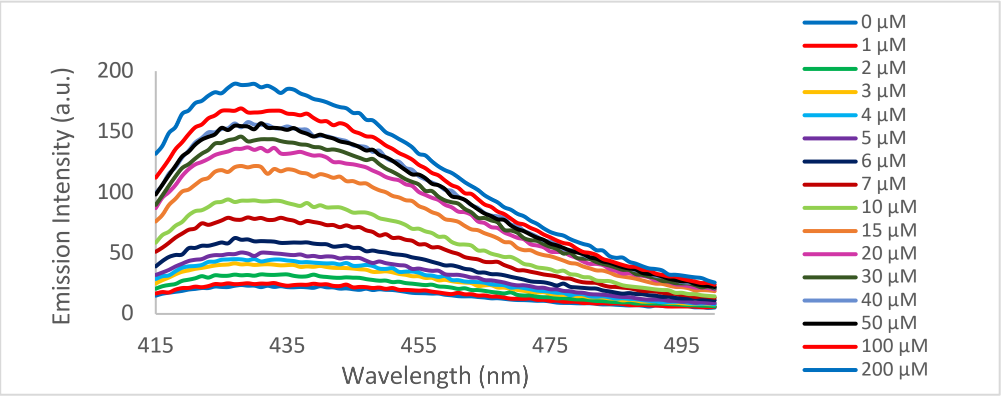

Figure S10 Competitive fluorescence titration added Phyt to a mixture of **CMP** (1 µM) and **CalixPyr** (4 µM) in 5 mM HEPES solution at pH 5.1 (λ_{ex} = 401 nm, λ_{obs} = 430 nm).

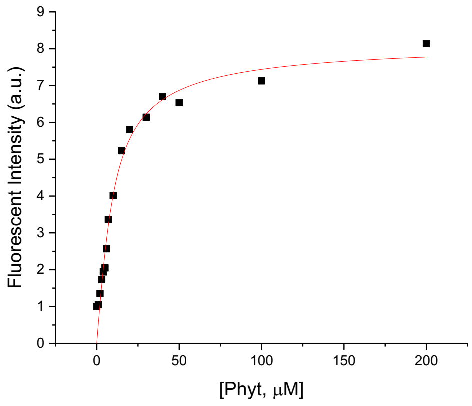

Figure S11 Competitive fluorescence titration added Phyt to a mixture of **CMP** (1 µM) and **CalixPyr** (4 µM) in 5 mM HEPES solution at pH 5.1 and fitted the plot of **CMP** fluorescence intensity to a 1:1 competitive binding model to give K_a for Phyt and **CalixPyr** = $(2.0 \pm 0.1)\times10^{6}$ M^{-1}. R^2 = 0.977.

ATP, ADP, AMP, and AP Titrations

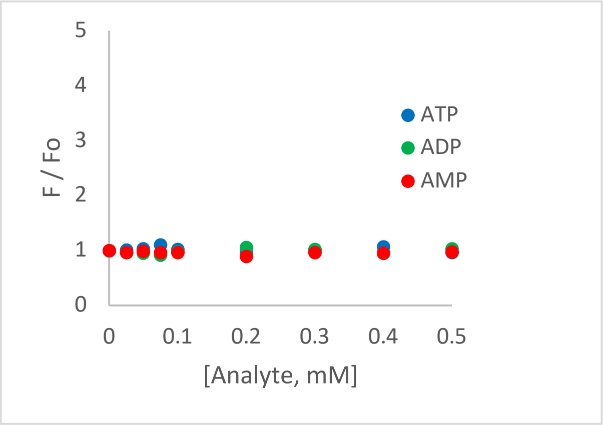

Figure S12 No change in **cCy5** emission intensity upon addition of ATP, ADP or AMP to a mixture of **CMP** (1 µM), **cCy5** (1 µM), and **CalixPyr** (4 µM) in 10 mM NaOAc solution at pH 7.2 at room temperature. λ_{ex} = 630 nm, λ_{obs} = 665 nm.

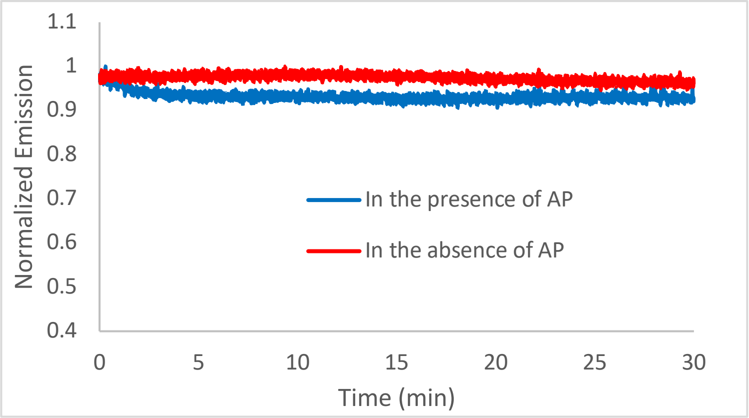

Figure S13 Negligible change in **cCy5** emission intensity during continuous fluorescence enzyme assay that added AP (1.5 U mL^{-1}, injected at 1 min) to a mixture of **CalixPyr** (4 µM), **CMP** (1 µM), **cCy5** (1 µM), and ATP (60 µM) in 10 mM NaOAc solution at pH 7.2, 37˚C. λ_{ex} = 630 nm, λ_{obs} = 665 nm.

References

1. E. A. Legenzov, N. D. A. Dirda, B. M. Hagen and J. P. Y. Kao, *PLoS one* **2015**, *10*, 1–18.
2. M. A. Spitsyn, V. E. Kuznetsova, V. E. Shershov, M. A. Emelyanova, T. O. Guseinov, S. A. Lapa, T. V Nasedkina, A. S. Zasedatelev and A. V Chudinov, *Dyes Pigments* **2017**, *147*, 199–210.
3. S. Shinoda, M. Tadokoro, H. Tsukube, S. Shinoda and R. Arakawa, *Chem. Commun*. **1998**, 2, 181–182.
